# A generalizable deep voxel-guided morphometry algorithm for the detection of subtle lesion dynamics in multiple sclerosis

**DOI:** 10.3389/fnins.2024.1326108

**Published:** 2024-01-25

**Authors:** Anish Raj, Achim Gass, Philipp Eisele, Andreas Dabringhaus, Matthias Kraemer, Frank G. Zöllner

**Affiliations:** ^1^Computer Assisted Clinical Medicine, Medical Faculty Mannheim, Heidelberg University, Mannheim, Baden Württemberg, Germany; ^2^Mannheim Institute for Intelligent Systems in Medicine, Medical Faculty Mannheim, Heidelberg University, Mannheim, Baden Württemberg, Germany; ^3^Department of Neurology, University Medical Centre Mannheim, Medical Faculty Mannheim, Heidelberg University, Mannheim, Baden Württemberg, Germany; ^4^Mannheim Center for Translational Neurosciences, Heidelberg University, Mannheim, Baden Württemberg, Germany; ^5^VGMorph GmbH, Mülheim an der Ruhr, Nordrhein-Westfalen, Germany; ^6^NeuroCentrum, Grevenbroich, Nordrhein-Westfalen, Germany

**Keywords:** multiple sclerosis, deep learning, brain MRI, voxel-guided morphometry, longitudinal change detection map, attention mechanism, generalizability

## Abstract

**Introduction:**

Multiple sclerosis (MS) is a chronic neurological disorder characterized by the progressive loss of myelin and axonal structures in the central nervous system. Accurate detection and monitoring of MS-related changes in brain structures are crucial for disease management and treatment evaluation. We propose a deep learning algorithm for creating Voxel-Guided Morphometry (VGM) maps from longitudinal MRI brain volumes for analyzing MS disease activity. Our approach focuses on developing a generalizable model that can effectively be applied to unseen datasets.

**Methods:**

Longitudinal MS patient high-resolution 3D T1-weighted follow-up imaging from three different MRI systems were analyzed. We employed a 3D residual U-Net architecture with attention mechanisms. The U-Net serves as the backbone, enabling spatial feature extraction from MRI volumes. Attention mechanisms are integrated to enhance the model's ability to capture relevant information and highlight salient regions. Furthermore, we incorporate image normalization by histogram matching and resampling techniques to improve the networks' ability to generalize to unseen datasets from different MRI systems across imaging centers. This ensures robust performance across diverse data sources.

**Results:**

Numerous experiments were conducted using a dataset of 71 longitudinal MRI brain volumes of MS patients. Our approach demonstrated a significant improvement of 4.3% in mean absolute error (MAE) against the state-of-the-art (SOTA) method. Furthermore, the algorithm's generalizability was evaluated on two unseen datasets (*n* = 116) with an average improvement of 4.2% in MAE over the SOTA approach.

**Discussion:**

Results confirm that the proposed approach is fast and robust and has the potential for broader clinical applicability.

## 1 Introduction

Multiple sclerosis (MS) is a chronic neurological disorder characterized by progressive loss of myelin and axonal structures in the central nervous system (Dal-Bianco et al., 2017). Serial MRI examinations of MS patients represent an important part of the diagnostic and monitoring workout of MS patients, including therapeutic decisions (Filippi et al., 2016; Dal-Bianco et al., 2017; Kaunzner and Gauthier, 2017). While the appearances of new and contrast-enhanced MS lesions are mostly related to clinical relapses, smoldering chronic active lesions, which are often not detectable in routine MRI scans, represent chronic inflammation and tissue destruction and may correlate with slow and chronic disease progression (Frischer et al., 2015). Accurate detection and monitoring of MS-related changes in brain structures are important background information for clinical management (Frischer et al., 2015). Evaluating subtle alterations across multiple examinations has become feasible to characterize disease evolution over time (Frischer et al., 2015). This includes fine analysis of white matter lesions, enlargement of the cerebrospinal fluid (CSF) compartment, and grey matter atrophy (Lewis and Fox, 2004).

Traditionally, the assessment of MS disease activity has primarily relied on the detection of new lesions (Filippi et al., 2016). Recently there has been an increasing interest in the detection of lesion activity including even subtle changes like smoldering lesions. There is a growing need for automated methods capable of generating complete maps quantifying structural brain tissue changes. Such methods are Voxel-Guided Morphometry (VGM) (Schormann and Kraemer, 2003) and deep VGM (Schnurr et al., 2020), where a neural network approximates a high dimensional deformation field for detecting changes in MS lesions in longitudinal MRI scans. The deep VGM approach by Schnurr et al. (2020) is fast, however, we intended to improve its robustness making it more applicable to a clinical setting. It is vital to develop a robust deep-VGM approach that is independent of the MRI system used. We aimed to develop a model that can effectively generalize to unseen datasets, allowing for fast, robust, and reliable monitoring of subtle MS disease activity.

In summary, this paper investigates a generalizable deep learning approach for Voxel-Guided Morphometry (VGM) map generation, such that it provides a generalizable tool for fast, accurate and automated analysis of subtle MS disease activity.

## 2 Materials and methods

### 2.1 Patient data

In this retrospective study, we analyzed two datasets of patients with multiple sclerosis (MS) from two different centers, following the 2010 diagnostic criteria by Polman et al. (2011). These datasets are referred to as Dataset A and Dataset B. Dataset A, which comprises 71 patients, is the same dataset utilized in the state-of-the-art method proposed by Schnurr et al. (2020). Dataset B consists of 97 patients. Every patient underwent two MRI examinations: one at baseline and a follow-up scan after a 12-month period. Patient demographics of these datasets are given in Table 1.

**Table 1:** Patient demographics of Dataset A and B.

**Property**	**Dataset A**	**Dataset B**
Gender (female:male)	64:13	70:27
Mean age (years)	37.67 ± 12.5	54.5 ± 14.6
PPMS	1	15
SPMS	4	19
RRMS	62	63
Mean disease duration (years)	5.71 ± 8.44	10 ± 14.32
Median EDSS (range)	2.0 (0–6.5)	3.5 (0–7.5)
Treatment (DMTs)	49/67	81/97

In the original study that compiled Dataset A, four patients were excluded, thus no patient demographics for these four patients were recorded. However, in this work, the imaging data of these patients were used. PPMS, primary progressive MS; SPMS, secondary progressive MS; RRMS, relapsing-remitting MS; EDSS, Expanded Disability Status Scale; DMTs, individually selected immune therapies.

For further validation, we procured an external public dataset comprising 19 patients from Carass et al. (2017). We call this Dataset C in our study. All patients were imaged with a high-resolution T1-weighted Magnetization Prepared Rapid Gradient Echo Image (MPRAGE) sequence. Please see the acquisition details in Table 2.

**Table 2:** Image acquisition characteristics for each dataset.

**Property**	**Dataset A**	**Dataset B**	**Dataset C**
Scanner	Magnetom Skyra, Siemens	Magnetom Allegra, Siemens	Unknown scanner, Philips
Field strength (T)	3.0	3.0	3.0
Sequence	T1-w MPRAGE	T1-w MPRAGE	T1-w MPRAGE
TR (ms)	1,900	2,080	10.3
TE (ms)	2.49	3.93	6
TI (ms)	900	1,100	835
Flip angle	9°	15°	8°
Spatial resolution (mm^3^)	0.94 × 0.94 × 2.00	1 × 0.98 × 0.98	0.82 × 0.82 × 1.17
Volume size (voxels)	256 × 256 × 70	160 × 240 × 256	256 × 256 × 120

MPRAGE, magnetization prepared rapid gradient echo.

### 2.2 Ground truth VGM generation

Voxel-Guided Morphometry (VGM) is a technique used for aligning 3D MRI images and generating maps that reveal global and regional changes in the brain between two sets of 3D MRI data collected at different time points. It utilizes T1-weighted MRI data. To initiate the process, high-quality brain masks are required, which can be generated using the FreeSurfer software package [refer to Segonne et al. (2004) for details]. The VGM process unfolds in four sequential steps:

1. Coarse linear alignment: In this initial step, VGM determines an affine transformation that maximizes the overlap between the brain masks of the two time points. This coarse linear alignment helps bring the images into initial alignment.
2. Inhomogeneity correction: To eliminate low-frequency bias in the images, a correction is applied by comparing the coarsely aligned images, as described in Lewis and Fox (2004).
3. Fine linear alignment: After inhomogeneity correction, a cross-correlation-based technique is employed to achieve finer alignment between the images. This step further refines the alignment achieved in the previous coarse alignment step.
4. The final step involves the application of a high-dimensional multiresolution full multigrid method. This step is crucial for capturing nonlinear deformations in the brain structures, allowing for comprehensive exploitation of information and effective image processing, as explained in Schormann and Kraemer (2003).

It's worth noting that typical computation times for these four steps on a CPU are ~4 min for steps (i) to (iii) and 7 min for step (iv).

In the ensuing stage, the VGM process orchestrates a guided movement for each voxel based on its grey value, aligning it from the source to the target image. The ultimate objective is to extract volume alterations for each voxel from the high-dimensional deformation field. The outcome is a map that assigns a quantified value to each voxel, indicating whether the corresponding brain region has undergone an increase or decrease in volume.

**Figure 1:**
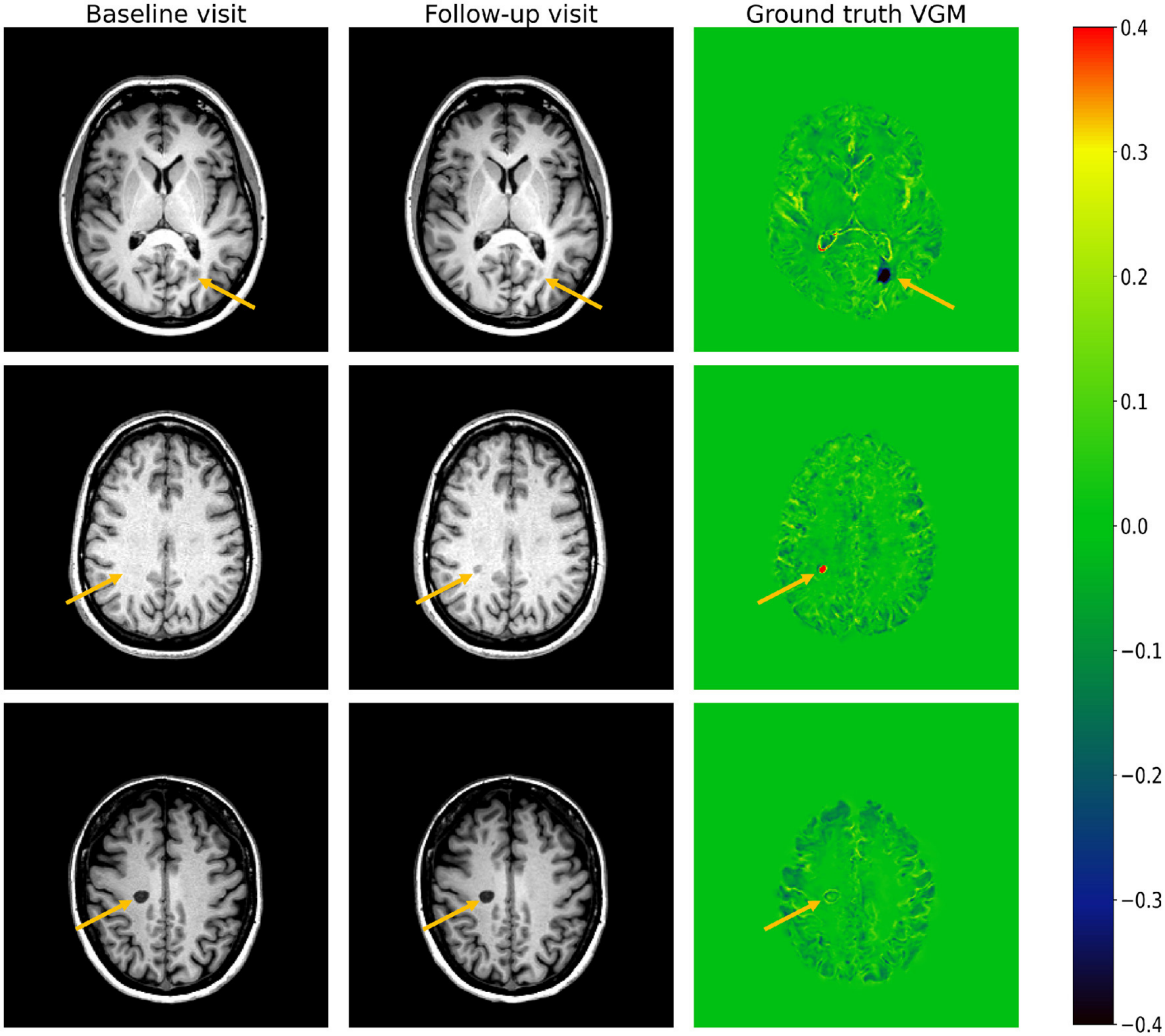
Three examples of VGM (ground truth) from a patient scan. **(Left)** Baseline image; **(middle)** follow-up image; **(right)** corresponding VGM map. The top slice depicts a lesion decreasing in volume (dark region). The middle slice VGM shows that one new lesion appeared in the follow-up visit (small bright-red region). The third slice shows a lesion with a very small change between the baseline and the follow-up visits. Arrows indicate the location of lesions in each case.

To illustrate the application of VGM, we provide an example case comprising a baseline image, a follow-up image, and the resulting VGM map in Figure 1. Initially designed for stroke data analysis (Schormann and Kraemer, 2003; Kraemer et al., 2004), recent research has demonstrated its efficacy in the context of multiple sclerosis (MS) (Kraemer et al., 2008; Fox et al., 2016; Weber et al., 2021, 2022). However, it is important to note that the clinical utilization of VGM is currently impeded by the relatively long computation time of ~11 min per case.

### 2.3 Image preprocessing

**Figure 2:**
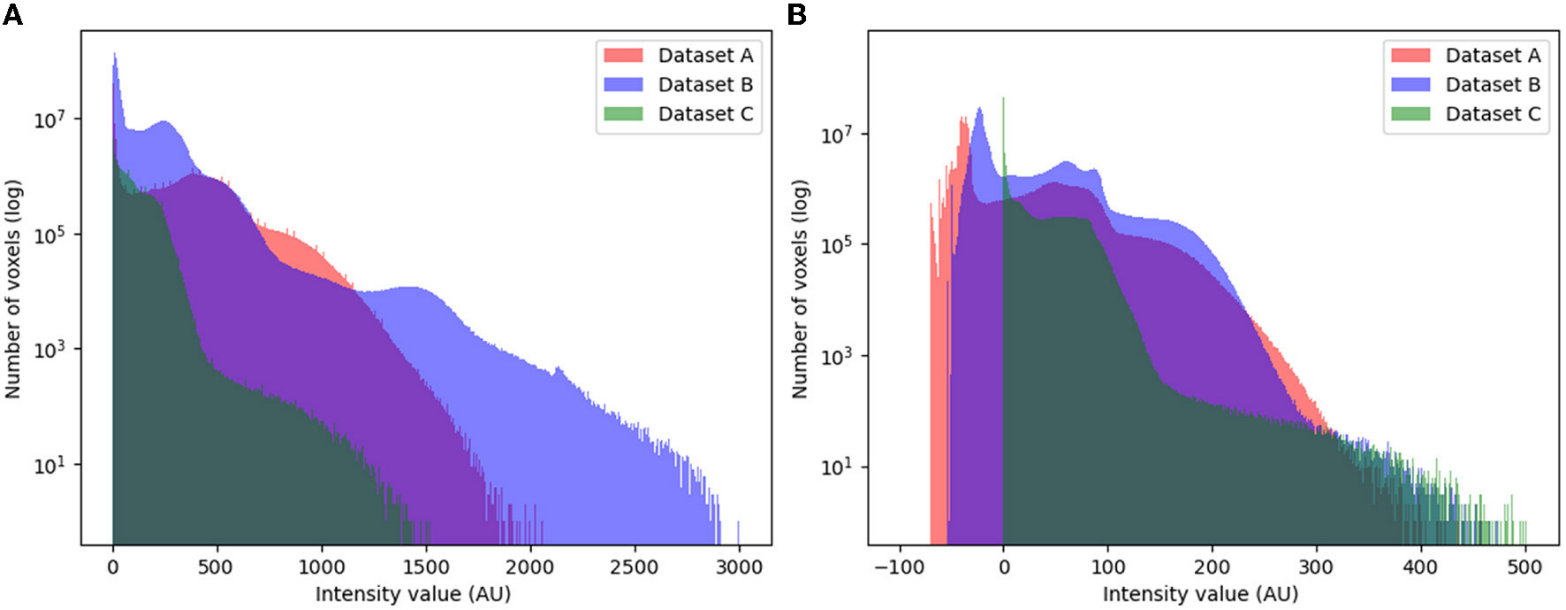
Histogram of image intensities of the original data distribution **(A)** and after Nyul normalization **(B)**. The *y*-axis scale is in logarithms (*x*-axis; AU, arbitrary units).

Initially, we perform histogram matching normalization using the Nyul normalization technique (Nyúl and Udupa, 1999). Specifically, we train the Nyul normalizer using Dataset A. Subsequently, we apply the trained normalizer to Datasets A, B, and C ensuring that the data distributions become identical. Following this step, we resample Dataset B and C to match the voxel spacing of Dataset A, which is 0.94 × 0.94 × 2.00 mm. Figure 2 illustrates the data distribution both before and after the application of histogram matching and resampling techniques. The visual comparison demonstrates a greater degree of similarity between the data distributions following the implementation of these techniques. Moreover, the calculated Wasserstein distances between the datasets A and B prior to and post-normalization are 62.77 and 25.48, respectively. Similarly, the distances between datasets A and C pre- and post-normalization are 100.19 and 31.39, respectively.

For the purpose of network training, we apply multiple preprocessing steps to the MRI volumes. These steps include bias correction, skull-stripping, and rigid registration (brain images of the two time points). Additionally, we adjust the image intensities to be confined within the interval of [0, 100] and then rescale them to the range of [−1, 1]. As for the VGM maps (labels), they are truncated to fit within the range of [−1, 1], while any values falling within the range of [−0.01, 0.01] are set to 0.

### 2.4 Attention mechanisms

In this work, we incorporate three attention modules into the U-Net architecture for improved VGM map prediction. These are described briefly in the following.

#### 2.4.1 Attention block from Attention U-Net

In their work, Oktay et al. (2018) introduced attention gates within the U-Net architecture. These attention gates serve as a mechanism to guide the network's decision-making process by selectively choosing relevant features while disregarding irrelevant ones. The authors achieved this by leveraging higher-level features as a guide to suppress trivial and noisy responses present in the lower-level skip connections. By incorporating attention gates, the network gains the ability to focus its attention on more informative features, thus enhancing its discriminative power and improving the overall performance of the U-Net model.

#### 2.4.2 Squeeze and excitation block

The SE block was introduced by Hu et al. (2018). This block is designed to enhance the representational power of convolutional neural networks (CNNs) by adaptively recalibrating feature maps. It consists of two main operations: squeezing and exciting. In the squeezing step, global spatial information is extracted by applying global average pooling to the input feature maps. This operation reduces the spatial dimensions of the feature maps. In the exciting step, the squeezed information is used to learn channel-wise dependencies and recalibrate the feature maps. This recalibration process enables the network to emphasize more informative channels and suppress less relevant ones, thereby improving the discriminative power of the network.

#### 2.4.3 Convolutional block attention module

The CBAM was first developed by Woo et al. (2018). It consists of two attention sub-modules: the channel attention module (CAM) and the spatial attention module (SAM). The CAM captures interdependencies between channels by adaptively recalibrating feature maps based on channel-wise information (similar to the SE block). It employs a combination of global average pooling and fully connected layers to compute channel attention weights. The SAM, on the other hand, captures spatial dependencies by adaptively highlighting informative spatial locations within feature maps. It utilizes the max-pooling and average-pooling operations followed by convolutional layers to generate spatial attention weights. By integrating both channel and spatial attention, the CBAM module enhances the discriminative power of CNNs and allows them to focus on salient features during image classification or object detection tasks.

### 2.5 Network architecture

We implemented three 3D U-Nets utilizing the aforementioned attention mechanisms to compute VGM maps from input volumes (Ronneberger et al., 2015; Raj et al., 2022). These U-Nets are equipped with residual and skip connections to facilitate the seamless flow of information and gradients. The encoder/decoder structure consists of five levels, with the first two levels comprising two convolution layers each, and the subsequent three levels consisting of three convolution layers each. The number of filters starts at 8 at the initial level and progressively increases to 128 at the bottom level. The VGM prediction map is generated through a final convolution layer of size 1 × 1 × 1, while all other convolutions employ 3 × 3 × 3 kernels. The inputs to the network consist of baseline and follow-up volumes.

We trained the following networks:

1. Attention U-Net: The U-Net architecture is enhanced with attention blocks in the decoder (Oktay et al., 2018).
2. SE-Attention U-Net: The U-Net architecture incorporates SE blocks in the encoder and attention blocks in the decoder (Hu et al., 2018; Raj et al., 2022).
3. CBAM-Attention U-Net: The U-Net architecture integrates CBAM blocks in the encoder and attention blocks in the decoder (Woo et al., 2018; Raj et al., 2022).

Furthermore, we compare our trained networks' performance with the baseline U-Net (Ronneberger et al., 2015) model obtained from the work of Schnurr et al. (2020).

**Figure 3:**
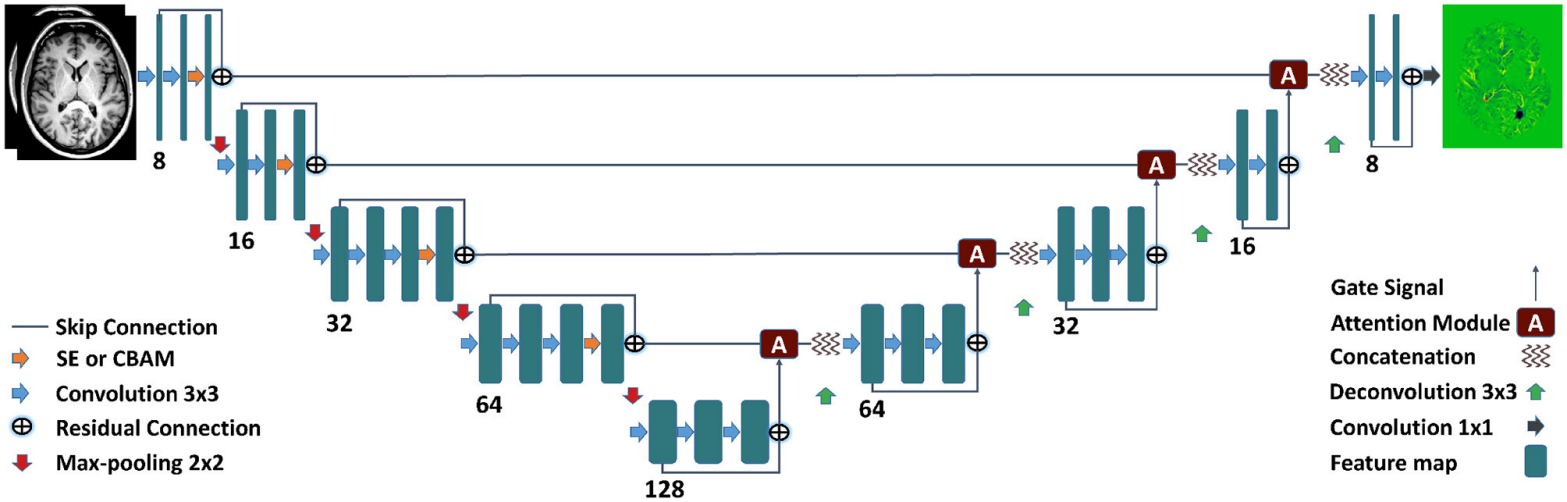
Proposed 3D U-Net incorporating attention mechanisms in the encoder and decoder parts. The attention mechanism from Oktay et al. (2018) is part of the decoder. The SE/CBAM blocks (Hu et al., 2018; Woo et al., 2018) are part of the encoder of the U-Net.

For a visual representation of these U-Net architectures, refer to Figure 3.

### 2.6 Loss function

The networks in our study are trained using a combination of mean absolute error (MAE) and gradient loss. The MAE loss, defined in Equation (1), calculates the average absolute difference between the predicted output Ŷ and the ground truth *Y*:


(1) $${ℒ}_{MAE}\left(Y,\hat{Y}\right)=\frac{1}{N}\sum_{i=1}^{N}|{\hat{y}}_{i}-y_{i}|,$$

To further improve the training process, we incorporate the combination of MAE and gradient loss, which is described in Equation (2).


(2) $$\begin{array}{l} {ℒ}_{Grad}\left(Y,\hat{Y}\right)=\frac{1}{N_{x}N_{y}N_{z}}\sum_{i,j,k}{\left(\left|y_{i,j,k}-y_{i-1,j,k}\right|-\left|{\hat{y}}_{i,j,k}-{\hat{y}}_{i-1,j,k}\right|\right)}^{2}\\ \;+\frac{1}{N_{x}N_{y}N_{z}}\sum_{i,j,k}{\left(\left|y_{i,j,k}-y_{i,j-1,k}\right|-\left|{\hat{y}}_{i,j,k}-{\hat{y}}_{i,j-1,k}\right|\right)}^{2}\\ \;+\frac{1}{N_{x}N_{y}N_{z}}\sum_{i,j,k}{\left(\left|y_{i,j,k}-y_{i,j,k-1}\right|-\left|{\hat{y}}_{i,j,k}-{\hat{y}}_{i,j,k-1}\right|\right)}^{2} \end{array}$$

The additional gradient loss term incorporates gradient information to guide the network's learning:


(3) $${ℒ}_{MAE+Grad}\left(Y,\hat{Y}\right)={ℒ}_{MAE}+\lambda \cdot {ℒ}_{Grad}$$

Where, ŷ_*i*_ and *y*_*i*_ represent the predicted and label voxel values, respectively. The total number of voxels in a batch is denoted by *N*, where *N*_*x*_, *N*_*y*_, and *N*_*z*_ represent the number of voxels along each dimension of the 3D MRI volume. Additionally, the parameter λ is set to one in Equation (2) to equally weight the two loss functions. We selected the $L_{MAE+Grad}$ loss function based on its demonstrated effectiveness in predicting VGM maps, as reported in Schnurr et al. (2020).

### 2.7 Training and implementation

In our study, we employed a training approach using 3D patches of size 128 × 128 × 32. The patches were sampled randomly, with the constraint that their centers lie within the brain mask, and were oriented along the transverse plane. Each training batch consisted of 8 samples.

For optimization, we utilized the Adam optimizer with a learning rate of 10^−3^. To mitigate overfitting, we applied L2 regularization with a weight of 10^−10^. Each network underwent training for a total of 860 epochs in a 5-fold cross-validation scheme. The 5-fold cross-validation was performed on Dataset A, which was split into train:validation:test sets with a ratio of 54:2:15 cases.

To assess the generalizability of our trained models, we applied them to Datasets B and C. This additional evaluation aimed to determine how well the models could perform on an independent, previously unseen dataset. It is also worth noting that for the baseline state-of-the-art method, we use the settings described in the baseline approach (Schnurr et al., 2020) to make predictions unless specified otherwise. In one scenario, we followed the same preprocessing steps as described in the baseline approach. In another case, we implemented our preprocessing steps, including intensity truncation in [0, 100], Nyul normalization, and resampling, for inference using the baseline model.

The neural networks were trained using Tensorflow 2.3.0 (Abadi et al., 2016) and Python 3.6.13, employing an Nvidia RTX A4000 as the GPU.

### 2.8 Evaluation

#### 2.8.1 Quantitative evaluation

To facilitate a more meaningful comparison, we utilize the same evaluation metrics described by Schnurr et al. (2020). These metrics allow us to assess the performance of our approach consistently.

The first metric we employ is the Mean Absolute Error (MAE), which quantifies the average absolute difference between the predicted and ground truth VGM map within the brain mask. This metric provides insight into the accuracy of the predicted VGM values at the voxel level. The second metric used for evaluation is the Structural Similarity Index (SSIM), a measure that assesses the similarity of structures between the predicted and ground truth VGM maps. The SSIM compares three components of images: luminance, contrast, and structure (Wang et al., 2004). This metric provides information about the overall structural preservation in the predicted VGM map compared to the ground truth. We further utilize the Dice Score (DSC), specifically for non-change regions within the brain mask. The DSC is calculated for voxel values falling within the range of [−0.01, 0.01] in both the predicted and ground truth VGM maps. This metric allows us to evaluate the similarity and overlap between these regions, further assessing the accuracy of the predicted VGM map. Finally, we perform a paired *t*-test to find a statistically significant difference (*p*-value < 0.05) between the results from the baseline and our proposed methods.

#### 2.8.2 Qualitative evaluation

Two expert neuroimagers (A.G., P.E.) performed a joined qualitative review by consensus. The predictions from our best-performing network were compared to conventional VGM maps (ground truth) and source T1-weighted data. The expert checked five patients from each dataset by comparing the predicted VGM and ground truth VGM in conjunction with the baseline and follow-up visits' MRI volumes. Based on the visual analysis, each patient's prediction in comparison to the ground truth VGM was categorized into four categories: 1. Missing information, where the prediction does not have enough details as compared to the ground truth, 2. loss of lesion to background contrast, where the lesion is lost to background and is not visible in prediction, 3. original result well-presented, where the prediction is of similar quality to the ground truth, and 4. additional lesion details offered, where the predicted VGM gives additional lesion information that might not be present in the ground truth.

## 3 Results

### 3.1 Quantitative results

**Figure 4:**
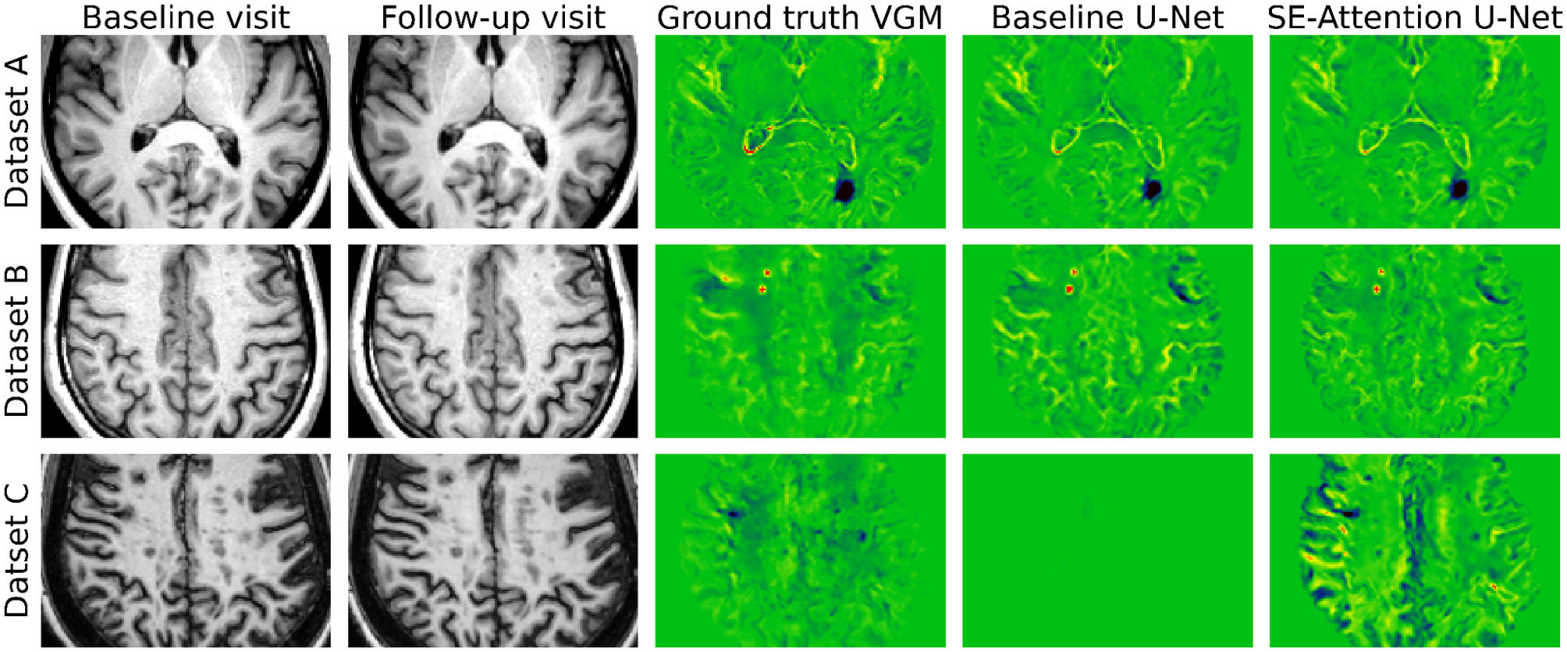
**(Top)** Dataset A, **(middle)** Dataset B, and **(bottom)** Dataset C. Qualitative results for each dataset from the baseline U-Net and our proposed approach with SE-Attention U-Net.

The results for each dataset are described in Table 3. For Dataset A, the CBAM-Attention U-Net attains the highest SSIM, DSC, and MAE of 0.9177, 0.9814, and 0.0335, respectively. In comparison, the baseline state-of-the-art method obtains SSIM, DSC, and MAE of 0.9139, 0.9800, and 0.0350, respectively. For Dataset B, the best SSIM, DSC, and MAE are 0.9416 (Attention U-Net), 0.9882 (CBAM-Attention U-Net), and 0.0337 (SE-Attention U-Net), respectively. Moreover, each network's metric for both Datasets A and B surpasses the corresponding metric for the baseline method. Furthermore, each of the proposed networks outperforms the baseline significantly in the case of Datasets A and B (*p*-value < 0.05). For Dataset C, the baseline method outperforms other networks in SSIM (0.9102) and MAE (0.0422) metrics. However, the DSC of the baseline (0.9287) is considerably lower than our networks' DSCs (best:0.9784). The poor DSC of the baseline method is due to the network outputting more values close to 0 in larger regions. The visual results in Figure 4 for Dataset C also confirm this, showing that although the baseline has slightly better metrics, the outputs are not useful for a physician.

**Table 3:** Results for each Dataset from networks trained on Dataset A only.

**Dataset**	**Network**	**SSIM ↑**	**DSC ↑**	**MAE ↓**
Dataset A	U-Net (baseline)	0.9139 ± 0.0216	0.9800 ± 0.0033	0.0350 ± 0.0112
	Attention U-Net	0.9172 ± 0.0213	0.9812 ± 0.0032	0.0338 ± 0.0107
	CBAM-Attention U-Net	0.9177 **±0.0212**	0.9814 **±0.0031**	0.0335 **±0.0105**
	SE-Attention U-Net	0.9172 ± 0.0212	0.9810 ± 0.0031	0.0337 ± 0.0106
Dataset B	U-Net (baseline)	0.9207 ± 0.0187	0.9816 ± 0.0034	0.0364 ± 0.0064
	Attention U-Net	0.9416 **±0.0143**	0.9881 ± 0.0023	0.0344 ± 0.0052
	CBAM-Attention U-Net	0.9406 ± 0.0144	0.9882 **±0.0023**	0.0351 ± 0.0053
	SE-Attention U-Net	0.9416 **±0.0143**	0.9880 ± 0.0022	0.0337 **±0.0052**
Dataset C	U-Net (baseline)	**0.9102** **±0.0377**	0.9287 ± 0.0093	**0.0422** **±0.0091**
	Attention U-Net	0.9097 ± 0.0288	0.9780 ± 0.0041	0.0465 ± 0.0087
	CBAM-Attention U-Net	0.9010 ± 0.0287	0.9784 **±0.0042**	0.0519 ± 0.0112
	SE-Attention U-Net	0.9091 ± 0.0291	0.9780 ± 0.0042	0.0459 ± 0.0086

The metrics for Dataset A are congregated from the results of 5-fold training. The metrics for Datasets B and C are calculated using an ensemble of five models that were trained on Dataset A. The baseline state-of-the-art method is outperformed in each metric for Datasets A and B by CBAM- or SE-Attention U-Net. However, for Dataset C, the SSIM and MAE of the baseline is higher than our approach. But as can be seen in Figure 4, the baseline method outputs VGM without any details. The MAE in this case is lower as most of the predicted values from the baseline method are close to 0. Further, the DSC of 0.92 is comparatively lower than the average 0.98 DSC from other methods. Numbers in bold signify the best metric for each dataset for each category. Underlined values depict significantly better metric in comparison to the corresponding baseline metric with *p*-value < 0.05. Up arrow signifies that higher value (number) is better, while down arrow signifies that lower values are better or desired.

**Figure 5:**
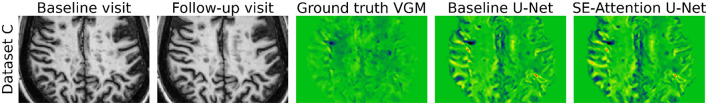
Visual result from the baseline U-Net for Dataset C (for the same patient from Figure 4) after applying our approach's preprocessing steps.

Furthermore, in Table 4, we depict results from the baseline method after applying the preprocessing methods from our approach (Section 2.3; i.e., truncation in [0, 100] range instead of baseline truncation in [200, 700] range, Nyul normalization, and resampling). In this case, the method improves on all metrics for Dataset B as compared to the corresponding result in Table 3. However, for Datasets A and C, SSIM and MAE numbers are lower in comparison. Noteworthily, the DSC of Dataset C improves and reaches 0.9778. After applying our preprocessing approach, the baseline network is able to produce meaningful predictions for Dataset C as depicted in Figure 5, suggesting that our preprocessing method is important for generalizability in this case.

**Table 4:** Results from the baseline method (ensemble) after applying image preprocessing described in our study.

**Dataset**	**Network**	**SSIM ↑**	**DSC ↑**	**MAE ↓**
Dataset A	U-Net (baseline)	0.9071 ± 0.0227	**0.9809** **±0.0034**	0.0379 ± 0.0120
Dataset B	U-Net (baseline)	**0.9396** **±0.0144**	**0.9879** **±0.0023**	**0.0348** **±0.0054**
Dataset C	U-Net (baseline)	0.9063 ± 0.0300	**0.9778** **±0.0042**	0.0467 ± 0.0097

As can be seen, the MAE for Dataset B improves, however, for Datasets A and C, it gets worse. The DSC for Dataset C increases by 6% as compared to the result in Table 3. The numbers in bold depict comparatively higher metrics to the corresponding baseline method from Table 3. Up arrow signifies that higher value (number) is better, while down arrow signifies that lower values are better or desired.

The prediction of each VGM map takes ~2.75 s. With the inclusion of preprocessing, the total time taken for VGM prediction is about 4 minutes [same as the SOTA method (Schnurr et al., 2020)].

### 3.2 Qualitative results

Table 5 shows the qualitative analysis result of five cases of each dataset that were visually analyzed by the experts. Since it achieved the best mean MAE score of 0.0377 across all the datasets, we selected SE-Attention U-Net as the best network for visual analysis. None of the predictions (0/15) showcased any missing information details. There is also no loss of lesion to background contrast (0/15) for any case in each dataset. Furthermore, all the analyzed predictions (15/15) show that they present the ground truth VGM well. Interestingly, one case in Dataset B (1/5) and three cases in Dataset C (3/5), show additional lesion details in comparison to the ground truth. However, for dataset A, the predicted VGMs do not offer (0/5) any additional lesion details in comparison to the ground truth.

**Table 5:** Visual inspection result by an expert neuro-radiologist for SE-Attention U-Net predictions compared to ground truth VGM, baseline and, follow-up MRIs.

**Dataset**	**Missing information**	**Loss of lesion to background contrast**	**Original result well-presented**	**Additional lesion detail offered**
Dataset A	0/5	0/5	5/5	0/5
Dataset B	0/5	0/5	5/5	1/5
Dataset C	0/5	0/5	5/5	3/5

Visual results are depicted in Figures 4, 5. In Figure 4, it can be seen that for Datasets A and B, the prediction from SE-Attention U-Net is similar to the ground truth, having dark [Dataset A (*n* = 1)] and bright spots [Dataset B (*n* = 2)] for changes in lesions in the same regions. For Dataset C, we show an example case for which our prediction offers better detail and more lesion information as compared to the ground truth. However, in this case, the baseline prediction is worse and does not show any lesion details. Interestingly, when we swap the baseline method's preprocessing with our preprocessing approach, the output is more informative and depicts VGM in higher quality (see Figure 5).

## 4 Discussion

We aimed to develop a generalizable approach to predict VGM maps for the longitudinal assessment of MS patients. Our work builds upon previous research (Schnurr et al., 2020) by addressing the crucial issue of generalizability. Some interesting aspects emerge from this work. The VGM maps help to detect subtle changes in lesions between baseline and follow-up visits. Our approach did calculate VGM maps in a short time and across three different datasets (2/3 unseen datasets) with high accuracy.

In the development process, we integrated advanced deep-learning techniques, image preprocessing, and careful model evaluation. Several steps were performed, that appeared useful and were able to improve the process incrementally. We began by describing the image preprocessing steps, which involved histogram matching normalization and voxel spacing resampling to ensure data consistency across different imaging centers. These preprocessing steps are crucial for enhancing the model's ability to generalize to diverse datasets. Our deep learning model is based on a 3D residual U-Net architecture, which incorporates attention mechanisms to highlight salient regions in the brain volumes. The application of attention mechanisms in MS lesion change detection is warranted as they have been shown to enhance lesion detection algorithms in previous works (Sarica and Seker, 2022; Sarica et al., 2023). To evaluate the effectiveness of our approach, we conducted extensive experiments using a dataset of 71 longitudinal MRI brain volumes of MS patients. We compared our model's performance to the SOTA method (Schnurr et al., 2020). Additionally, we evaluated the generalizability of our model on two unseen datasets, to test its robustness and potential for broader clinical applicability.

In our approach, we show that across all the datasets, the method attains SSIM and DSC higher than 0.91 and 0.98, respectively. Furthermore, the differences in the MAEs for each dataset are not considerably high. The current state-of-the-art (baseline) for predicting VGM maps using deep learning, as proposed by Schnurr et al. (2020), involves the implementation of a U-Net model without attention mechanisms. This baseline method does not adequately address the generalizability across different scanners and sites, a limitation our approach seeks to overcome. Employing the trained model from Schnurr et al. (2020) on Dataset A, we obtained an SSIM of 0.9139, a DSC of 0.9800, and an MAE of 0.0350 (Table 3)^1^. In comparison, each of our proposed networks surpassed the state-of-the-art result for Dataset A. Similarly, we extended the baseline to Datasets B and C and found that our approach outperforms the baseline for Dataset B. However, for Dataset C, the baseline attained higher SSIM and MAE scores (Table 3). In contrast, the baseline DSC in this case is considerably lower (0.92 v/s 0.97). When visually analyzed (Figure 4), we found that the baseline result could not replicate the ground truth VGM and contained values very close to 0 (therefore lower DSC), and hence it attained better MAE in comparison. In this case (Dataset C), the numbers did not reflect the results visually and were deemed not useful in a clinical setting. However, when we applied our preprocessing approach (i.e., Nyul normalization + truncation in [0, 100] range), the baseline method yielded a much more convincing VGM map as seen in Figure 5. This suggests that the preprocessing method is key to the generalizability of deep VGM maps. The resulting average MAE is 0.0467 (Table 4), which is worse than our SE-Attention U-Net. Furthermore, we found that for Datasets B and C, the predicted VGM maps could offer extra lesion details in comparison to the ground truth maps without the loss of important information in 4/15 cases (Table 5). This also suggests that the ground truth VGM maps of Dataset A are of higher quality and training on them could help the network learn high-quality features.

Moreover, there exist multiple automated new lesion segmentation algorithms based on deep learning (Andresen et al., 2022; Ashtari et al., 2022; Basaran et al., 2022; Hitziger et al., 2022; Kamraoui et al., 2022; Sarica and Seker, 2022; Schmidt-Mengin et al., 2022; Commowick et al., 2023). These methodologies primarily leverage the MSSEG-2 dataset (Commowick et al., 2021), encompassing FLAIR images from baseline and follow-up visits for each patient, either with or without the integration of synthetic data. Notably, these approaches focus on segmenting new lesions during follow-up visits. In contrast, our proposed approach distinguishes itself by its fundamental objective: quantifying the change in lesion activity between baseline and follow-up measurements. Unlike the aforementioned methods, which aim to delineate new lesions, our approach offers a distinctive perspective, providing quantitative insights into the variations in lesion size between visits. This ability to quantitatively showcase the decrease or increase in lesion activity enhances the depth and specificity of our methodology in assessing lesion dynamics over time.

Nevertheless, methodologies analogous to ours have been proposed in the literature, specifically targeting the identification of change maps in MS patients between baseline and follow-up examinations. Dufresne et al. (2022) presented an algorithm that concurrently optimized image registration and local intensity change detection within FLAIR volumes. Cheng et al. (2018) computed lesion changes utilizing T1, T2, and FLAIR sequences. Their approach involved estimating a dissimilarity map between two visits and subsequently incorporating logistic regression with neighborhood information and local texture descriptors. It is noteworthy that a direct comparison between our approach and these existing methodologies is challenging due to the fact that both aforementioned approaches utilize lesion segmentation maps as the ground truth for evaluation. In contrast, our study necessitates expert annotation and an optimal threshold for generating lesion maps (derived from VGM maps) to facilitate segmentation evaluation. Such a comparative task extends beyond the scope of the current work.

Addressing the challenge of MRI data heterogeneity across sources is essential for the widespread adoption of deep learning-based tools in clinical practice, and our model's independence from MRI sources demonstrates its potential as a versatile clinical asset. Furthermore, the high accuracy and generalizability of our deep learning approach hold great promise for clinical practitioners, as it offers a valuable tool for detecting and monitoring subtle changes in MS lesions, facilitating more informed treatment decisions. The ability to identify even the most discreet changes in brain structures could significantly impact the clinical management of MS patients, potentially leading to earlier interventions and improved patient outcomes.

Nonetheless, our approach has a few limitations. Firstly, it needs accurately registered brain volumes for baseline and follow-up visits. If the registration is of low quality, then the VGM maps will be less accurate and might display less precise information. However, we found that all the cases in our study were registered with high quality. Another limitation could be the (partial) loss of lesion when resampling datasets to have the same spacing as Dataset A. Dataset A has anisotropic spacing where the slice thickness is 2 mm. Resampling a higher-resolution MRI volume to a 2 mm slice thickness could result in partial volume effects, i.e., loss of some detail. In this case, it would mean that the VGM maps might be less precise in detecting some subtle lesion changes.

In future work, we will analyze if the VGM maps can be produced from a single scan of MS patients with a comparison scan from an age- and sex-matched group of healthy individuals for detecting lesions. Furthermore, we would also test our algorithm for detecting structural changes in other neurological diseases such as stroke, neurodegenerative diseases, or brain tumors.

In conclusion, we present a generalizable approach that can produce VGM maps in a fast and robust manner across datasets from various sources. Our algorithm can be helpful in detecting subtle lesion changes in brains of MS patients.

## Data availability statement

The data analyzed in this study is subject to the following licenses/restrictions: the data presented in this study are available on reasonable request from the corresponding author. The Datasets A and B are not publicly available due to data protection and patient privacy regulations. The Dataset C analyzed for this study can be found in the data download link at https://smart-stats-tools.org/lesion-challenge-2015. Requests to access these datasets should be directed to anish.raj@medma.uni-heidelberg.de.

## Ethics statement

The studies involving humans were approved by Institutional Ethics Committee of the Universitätsmedizin Mannheim. The studies were conducted in accordance with the local legislation and institutional requirements. Written informed consent for participation was not required from the participants or the participants' legal guardians/next of kin in accordance with the national legislation and institutional requirements.

## Author contributions

AR: Conceptualization, Data curation, Formal analysis, Investigation, Methodology, Software, Visualization, Writing – original draft, Writing – review & editing. AG: Conceptualization, Formal analysis, Investigation, Supervision, Validation, Visualization, Writing – review & editing. PE: Data curation, Writing – review & editing, Validation, Visualization. AD: Data curation, Resources, Writing – review & editing, Software, Validation. MK: Resources, Validation, Writing – review & editing, Data curation. FZ: Conceptualization, Funding acquisition, Project administration, Resources, Supervision, Validation, Writing – original draft, Writing – review & editing.

## Footnotes

^1^In our study, discrepancies were observed in the metric values for the baseline method when compared to those reported in Schnurr et al. (2020). Despite employing the same trained models and adhering to the preprocessing protocol detailed in their paper, the variation in results could be attributed to small details in their methodology that were not described in their paper. Additionally, the divergence in outcomes may also stem from variances in the versions of the libraries used between the two studies.
